# Rectal Cancer Disparities Among the American Indian/Alaskan Native Populations

**DOI:** 10.1002/cam4.70892

**Published:** 2025-04-19

**Authors:** Broc S. Kelley, Cibele B. Carroll, John M. Hampton, Margaret R. Walker, Syed Nabeel Zafar, Dana Hayden, Andrea Schiefelbein, Roberto J. Vidri, Bret Benally Thompson, Noelle K. LoConte

**Affiliations:** ^1^ Department of Medicine, Division of Hematology, Oncology and Palliative Care University of Wisconsin School of Medicine and Public Health Madison Wisconsin USA; ^2^ University of Wisconsin Carbone Cancer Center Madison Wisconsin USA; ^3^ Division of Surgical Oncology Department of Surgery University of Wisconsin School of Medicine and Public Health Madison Wisconsin USA; ^4^ Division of Colorectal Surgery Department of Surgery University of Wisconsin School of Medicine and Public Health Madison Wisconsin USA; ^5^ Morgridge Institute for Research Madison Wisconsin USA; ^6^ Department of Surgery, Division of Surgical Oncology and Endocrine Surgery University of North Carolina School of Medicine Chapel Hill North Carolina USA

**Keywords:** American Indian or Alaska Native, colorectal cancer, health status disparities, minority health, rectal cancer

## Abstract

**Purpose:**

Recent work noted lower overall survival (OS) in American Indian/Alaskan Native (AI/AN) individuals diagnosed with colon cancer compared with non‐Hispanic White (NHW) individuals. Rectal cancer demographic profiles at diagnosis and survival outcomes have not been reported. We sought to identify differences in rectal cancer diagnosis and outcomes between AI/AN and White populations.

**Methods:**

White and AI/AN patients aged 18 or older, diagnosed between 2004 and 2020 with rectal adenocarcinoma were identified within the National Cancer Database (NCDB). Unadjusted and adjusted analyses were used to evaluate demographic and clinical standardized differences (stddiff) between AI/AN and White patients. Survival analyses of those diagnosed with locally advanced rectal cancer (Stage II/III) were performed using the Kaplan–Meier methods and multivariate Cox‐proportional hazards modeling.

**Results:**

176,341 eligible cases were identified: 0.6% were AI/AN (*N* = 992) and 99.4% White (*N* = 175,349). Compared to the White population, AI/AN patients were younger at diagnosis (mean age 59.9 vs. 64.5 years; stddiff = 0.36) and had more advanced stage disease (44.8% vs. 43.7%; stddiff = 0.15). A higher percentage of AI/AN resided in the areas of the lowest median income (35.5% vs. 15.1%; stddiff = 0.62) per zip code, rural (9.9% vs. 2.2%; stddiff = 0.65), and used Medicaid as their primary payor (14.3% vs. 6.2%; stddiff = 0.63). Adjusted analyses suggest the AI/AN group has an increased hazard of death compared with the White population (HR, 1.14; 95% CI, 1.05–1.25; *p* = 0.003).

**Conclusions:**

AI/AN patients with rectal cancer have a younger age and a more advanced stage at diagnosis. AI/AN race is associated with lower OS compared to White patients in multivariable analyses. Future efforts should focus on increasing colorectal cancer screening and access to treatment for AI/AN populations in an attempt to improve survival outcomes.

## 1 Introduction

North American Indigenous and Alaskan Native populations represent distinct tribal nations with rich histories deeply embedded within the fabric of North America [1, 2, 3]. These communities, although diverse in culture and language, share common threads in their relationship to the land and their experiences of colonization and displacement [3, 4]. With an estimated 9.7 million North American Native individuals residing in the U.S. in 2020, their presence spans across states, with Alaska, Oklahoma, New Mexico, and South Dakota housing significant populations [3]. The unique health‐related challenges faced by these communities are complex and multifaceted [3, 4].

Observational studies have consistently highlighted disparities in health outcomes for Indigenous populations, with notable inequities in cancer incidence and mortality [3, 4, 5, 6, 7, 8]. Colorectal cancer (CRC) poses a significant burden, being the second leading cause of cancer deaths among North American Native populations, behind lung cancer [3, 8]. National trends have underscored an increase in colorectal cancer incidence among younger individuals [3, 9], a trend not fully explained but postulated to be due to the conditions in which Indigenous people live and work [10]. These conditions are determined by centuries of discriminatory practices and policies, implemented because European colonization [4], that forced many communities to experience socioeconomic disadvantages over the years when compared to White individuals of European ancestry [10]. Ultimately, the structure of prolonged socioeconomic disadvantages affects lifestyle choices, increases exposure to environmental carcinogens [11], and prints its effects through epigenetic changes [10, 12].

Colon and rectal adenocarcinoma share many commonalities such as risk factors, initiation through mucosal cell proliferation and polyp growth, screening, and early detection strategies. However, colon and rectal cancer differ in treatment and outcomes [13]. Rectal cancer treatment has specificities related to its embryological origin and pelvic location in proximity to urogenital organs [13]. Prior work reported on differences in the age of diagnosis and survival rates among American Indian/Alaskan Native (AI/AN; term specifically used within the National Cancer Database [NCDB]) and non‐Hispanic White patients treated for colon cancer [5]. This study uses data from the NCDB to elucidate the demographic profile and treatment outcomes of AI/AN individuals diagnosed with rectal cancer. We hypothesize that there are differences in rectal cancer presentation at diagnosis and overall survival (OS) between White and AI/AN individuals diagnosed with locally advanced rectal cancer (LARC).

## 2 Methods

### 2.1 Data Source, Study Population, and Covariate Selection

We performed a retrospective cohort analysis of AI/AN individuals diagnosed with rectal cancer, drawing from the NCDB. The NCDB aggregates treatment data from about 1500 Commission on Cancer (CoC)‐accredited hospitals [14, 15], approximating 70% of newly diagnosed cancers in the United States. Although a comparison with the United States Cancer Statistics (USCS) data for the years 2012–2014 showed that NCDB covered approximately 41% of the United States' AI/AN patients with cancer [15]. Our examination is limited to rectal cancer cases diagnosed from 2004 to 2020, with each case associated with a single CoC treatment facility, and with the following adenocarcinoma histology codes 8140, 8141, 8143, 8144, 8145, 8147, 8150, 8154, 8160, 8161, 8163, 8190, 8200, 8201, 8210, 8211, 8213, 8220, 8221, 8230, 8243, 8250, 8254, 8260, 8261, 8310, 8320, 8323, 8380, 8401, 8410, 8440, 8460, 8470, 8490, 8500, 8503, 8510, 8265, 8507 [14]. The NCDB is a joint project of the CoC of the American College of Surgeons and the American Cancer Society [16]. The CoC's NCDB and the hospitals participating in the CoC's NCDB are the source of the de‐identified data used herein [16]; they have not verified and are not responsible for the statistical validity of the data analysis or the conclusions derived by the authors. This study was deemed exempt from review by the University of Wisconsin Health Sciences Institutional Review Board.

In NCDB, the variable of race is reported by hospital systems and ideally collected from patient self‐reported answers. Race was the primary variable of interest, specifically comparing the AI/AN population against the reference White population. A brief note on terminology: through this study we refer to the North American and Alaskan Native populations as American Indian/Alaskan Native (AI/AN) given this is the specific terminology from the NCDB, but we acknowledge that this (“Indian”) is an outdated term. In addition, the term AI/AN is more reflective of ethnicity, a population sharing culture, language, ancestry, and practices, rather than race, which is how it is used in the NCDB. We acknowledge that race is a social construct that groups individuals on the basis of phenotypic characteristics, and the term American Indian/Alaska Native does not capture the heterogeneity of the distinct tribal nations [4]. The White population was chosen as the reference population due to their historical privilege in the United States. Relevant covariates were collected for analysis, including sex, age, stage at diagnosis (as classified by the American Joint Committee staging system) [17, 18], treatment facility type, treatment facility location, population density, income, educational attainment, primary insurance, Charlson–Deyo comorbidity score [19], and cancer treatment modalities [16, 17]. The variable of analytic stage was utilized in this study as it reports the pathologic stage or clinical stage group in case the pathologic stage is not reported [17]. Stage 0 was excluded from the analysis due to its overall good prognosis. Therefore, all other stages (I–IV and Unknown) were included in descriptive analysis.

Survival analysis was limited to LARC—defined as Stages II and III in this population. This is because of the LARC population's unique multidisciplinary needs regarding treatment and potential for cure. In addition, this limits distortion of survival by excluding low and high stages. To further guide the survival analysis, the research team created a directed acyclic graph [20] with the variables included in the study (Figure S1). In this graph, race was considered the exposure and the OS the outcome of interest. This way, other known mediating pathways were controlled to assess the direct effects of race on survival [20].

### 2.2 Statistical Analysis

Demographic variables and treatment outcomes of AI/AN individuals were compared to their White counterparts. Chi‐square and ANOVA tests were utilized for categorical and continuous variables, respectively. Box plots and bar graphs were utilized to visually analyze the age at diagnosis and the distribution of analytic stages between AI/AN and White populations, with the NCDB analytic stage employed due to a high proportion of unknown clinical and pathologic stage data [17]. For descriptive analysis, standardized difference (stddiff), calculated as per Bayoumi and Austin, offered a scale‐free measure to assess differences between AI/AN and White populations, considering the magnitude of these differences beyond mere statistical significance [21, 22, 23, 24, 25]. The standardized difference score is utilized to measure the differences between groups independently of the influence of sample size [21]. For continuous variables, the standardized difference takes into account the means and variances of the groups being compared, whereas for categorical variables, it is on the basis of the prevalence of the variables among groups [22]. Considering the large sample size available in NCDB, the differences among groups were reported in standardized differences rather than with *p*‐values. In this manuscript, the standardized differences are reported for the variable (labeled as group) and for the presence of specific categories within each variable (labeled as category).

OS was defined as the length of time elapsed between the date of diagnosis and the date of last contact or death. Kaplan–Meier survival curves and log‐rank tests delineated survival differences in an unadjusted population, stratified by age at diagnosis (before and after age 45, the typical recommended age for screening initiation). A multivariable Cox‐proportional hazard model was created to further assess survival. The included covariates were age, sex, stage, comorbidities, CoC facility type, insurance status, radiation therapy, and CoC facility location. To combat immortal time bias, two specific time‐varying covariates were included—namely surgery and chemotherapy received. Each patient undergoing these interventions was split into multiple instances, with the first (no treatment) censored at the time of chemotherapy received or surgery performed (whichever came later). The second instance (treatment received) was then censored at the time of death or last contact as usual. This allowed direct survival time comparison of those with and without treatment intervention, accounting for immortal time bias. Proportional hazard assumption was assessed through Shoenfeld residuals and log‐log plots given the extensive number of cases. We noted borderline violation of the proportional hazard assumption with sex and stage variables, but given the clinical significance of these metrics, they were included in the final model. Similarly, treatment modalities seemingly violated the proportional hazard assumption. However, sensitivity testing using strata for treatment modalities (a way to account for assumption violation) did not result in meaningful differences in the final model or interpretation; thus, the full model (including treatment) was used. All models were rectal cancer‐specific. STATA version 18 and SAS version 9.4 were utilized, with a statistical significance threshold set at *p* < 0.05. Cases with incomplete demographic, clinical, or treatment information were accounted for by including “unknown” categories.

## 3 Results

From 2004 to 2020, a total of 233,637 patients with rectal adenocarcinoma were identified within the data. The following cases were excluded from the descriptive analysis: (1) 35,553 patients not identified as AI/AN or White, (2) 12,985 patients diagnosed in the year 2020 who did not have vital status information available in the database, (3) 8740 patients with Stage 0 rectal cancer, and (4) 18 patients with incomplete (missing) vital status or censoring information. For the survival analysis, the population was further limited to LARC. An additional 44,033 patients with Stage I, 30,939 patients with Stage IV, and 17,691 patients with unknown stage cancers were excluded (Figure 2).

### 3.1 Patient Population

**FIGURE 1:**
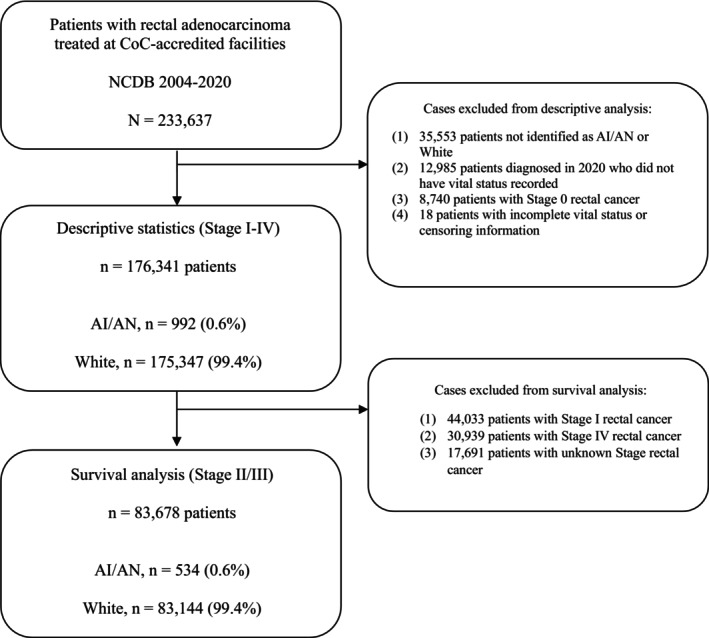
Flowchart with inclusion and exclusion criteria for data analysis. AI/AN, American Indian Alaska Native; NCDB, National Cancer Database.

A cohort of 176,341 cases of rectal adenocarcinoma was analyzed (Table 1). Within this population, 992 (0.6%) patients were identified as AI/AN, and the remaining 175,349 (99.4%) as White (Figure 1). AI/AN individuals disproportionately resided in areas of the lowest median income (35.5% vs. 15.1%; stddiff = 0.62) defined by census track data as under $40,277 per year. In addition, a significant proportion of AI/AN patients lived in rural areas (9.9% versus 2.2%; stddiff = 0.7). They also lived in areas that had a higher proportion of individuals not completing high school (30.5% vs. 18%; stddiff = 0.4) per zip code. Regarding insurance, the proportion of AI/AN patients with Medicaid was higher than that of White patients (14.3% vs. 6.2%; stddiff = 0.63).

**TABLE 1:** Demographic characteristics of AI/AN and White patients with rectal cancer on NCDB 2004–2020.

	AI/AN, *n* = 992	White, *n* = 175,349	Standardized difference	
Age mean(±SD)	59.86 (12.33)	64.52 (13.66)	0.36	
Sex *n* (%)			Group	Category
Female	378 (38.1)	69,248 (39.5)	0.03	
Male	614 (61.9)	106,101 (60.5)		
AJCC stage *n* (%)				
Stage 1	200 (20.2)	43,833 (25)	0.15	0.12
Stage 2	261 (26.3)	37,245 (21.2)		0.12
Stage 3	273 (27.5)	45,899 (26.2)		0.03
Stage 4	171 (17.2)	30,768 (17.6)		0.01
Unknown	87 (8.8)	17,604 (10)		0.04
Charlson‐Deyo score *n* (%)				
Score 0	721 (72.7)	132,833 (75.8)	0.08	0.07
Score 1	188 (19)	29,656 (16.9)		0.05
Score 2	49 (4.9)	8247 (4.7)		0.01
Score 3+	34 (3.4)	4613 (2.6)		0.05
Facility type^a^ *n* (%)				
Community cancer program	104 (10.5)	13,368 (7.6)	0.21	0.10
Comprehensive community cancer program	450 (45.4)	71,280 (40.7)		0.10
Academic/research program	264 (26.6)	52,224 (29.8)		0.07
Integrated network cancer program	126 (12.7)	32,614 (18.6)		0.16
Unknown	48 (4.8)	5863 (3.3)		
Urban/rural *n* (%)				
Metro	498 (50.2)	137,694 (78.5)	0.65	0.62
Urban	361 (36.4)	27,786 (15.9)		0.48
Rural	98 (9.9)	3826 (2.2)		0.33
Unknown	35 (3.5)	6043 (3.5)		
Income median quartile per zip code/2016 *n* (%)				
<$40,227	352 (35.5)	26,413 (15.1)	0.62	0.48
$40,227–$50,353	214 (21.6)	36,795 (21)		0.01
$50,353–$63,332	164 (16.5)	38,433 (21.9)		0.14
≥ $63,333	121 (12.2)	54,128 (30.9)		0.47
Unknown	141 (14.2)	19,580 (11.2)		
Population without HSD per zip code/2016 *n* (%)				
≥ 17.6%	303 (30.5)	31,477 (18)	0.40	0.30
10.9%–17.5%	244 (24.6)	41,417 (23.6)		0.02
6.3%–10.8%	192 (19.4)	45,174 (25.8)		0.15
< 6.3%	115 (11.6)	38,056 (21.7)		0.27
Unknown	138 (13.9)	19,225 (11)		0.08
Insurance *n* (%)				
Not insured	32 (3.2)	6561 (3.7)	0.63	
Private insurance	308 (31.1)	71,077 (40.5)		0.20
Medicaid	142 (14.3)	10,842 (6.2)		0.27
Medicare	338 (34.1)	81,345 (46.4)		0.25
Other government (IHS)	150 (15.1)	2426 (1.4)		0.52
Unknown	22 (2.2)	3098 (1.8)		
Surgical treatment *n* (%)				
Surgery	703 (70.9)	125,499 (71.6)	0.06	0.02
No surgery	281 (28.3)	49,223 (28.1)		0.01
Unknown	8 (0.8)	627 (0.4)		0.06
Chemotherapy *n* (%)				
Chemotherapy	662 (66.7)	111,793 (63.8)	0.07	0.06
No chemotherapy	302 (30.4)	59,323 (33.8)		0.07
Unknown	28 (2.8)	4233 (2.4)		0.03
Radiation therapy *n* (%)				
Radiation	155 (15.6)	23,136 (13.2)	0.12	0.07
No radiation	199 (20.1)	42,877 (24.5)		0.11
Unknown	638 (64.3)	109,336 (62.4)		0.04
Regional lymph nodes examined *n* (%)				
≤ 12	605 (61)	106,499 (60.7)	0.02	0.01
> 12	369 (37.2)	66,024 (37.7)		0.01
Unknown	18 (1.8)	2826 (1.6)		0.02

Abbreviations: HSD, high school diploma; IHS, Indian Health Services; NCDB, National Cancer Database.

^a^ Facility Type definition: Community Cancer Program facilities receive between 100 and 500 newly diagnosed cancer cases per year. Comprehensive Community Cancer Program facilities receive greater than five hundred newly diagnosed cancer cases per year. Academic/Research Program facilities participate in postgraduate medical education in at least four areas and receive more than five hundred newly diagnosed cancer cases per year. Integrated Network Cancer Program facilities belong to an organization and are overseen by a centralized governance structure.

### 3.2 Age and Stage at Diagnosis

**FIGURE 2:**
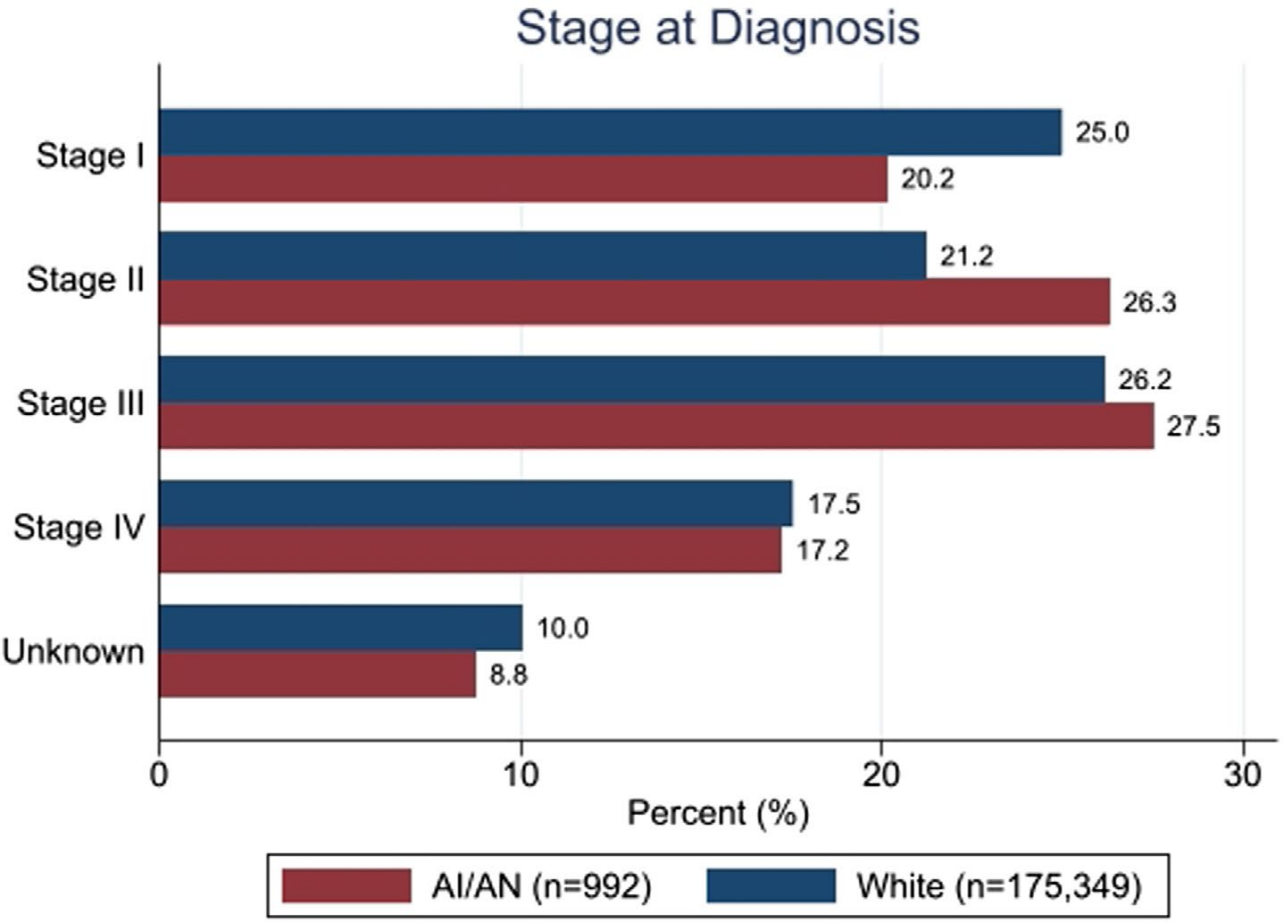
Bar chart with AJCC staging by race. Numbers at the end of bars in black represent the percentage of Stage I–IV in each group. AI/AN, American Indian/Alaska Native.

AI/AN patients were diagnosed at a younger mean age of 59.9 years, compared to 64.5 years for White patients (stddiff = 0.36) (Figure S2); they also more frequently presented with advanced stages of cancer (44.8% in Stages III or IV vs. 43.7%, stddiff = 0.15). Figure 2 shows the distribution of AJCC stages among AI/AN and White patients.

### 3.3 Rectal Cancer Treatment

AI/AN patients were more often treated at comprehensive community cancer program facilities compared with White patients (45.4% vs. 40.7%; stddiff = 0.21). Proportionally more White patients were treated at academic/research programs and integrated network cancer programs (29.8% and 18.6%) compared with their AI/AN counterparts (26.6% and 12.7%). AI/AN patients were also more likely to receive chemotherapy (66.7% vs. 63.8%; stddiff = 0.07) and radiation therapy (15.6% vs. 13.2%; stddiff = 0.11) than White patients. No differences were observed regarding the receipt of surgical treatment (70.9% vs. 71.6%) or the number of regional lymph nodes examined (37.2% vs. 37.7% had > 12 lymph nodes examined).

When stratified by stage of disease: 92.0% of White patients versus 90.5% of AI/AN patients had surgery for Stage I disease (*p* = 0.32), 81.2% of White patients versus 82.4% of AI/AN patients had surgery for Stage II or III disease (*p* = 0.17), and 30.6% of White patients versus 26.9% of AI/AN patients had surgery for Stage IV disease (*p* = 0.51). Regarding chemotherapy, 36.1% of White patients versus 40.2% of AI/AN patients had chemotherapy for Stage I disease (*p* = 0.03), 80.2% of White patients versus 80.5% of AI/AN patients had chemotherapy for Stage II or III disease (*p* = 0.99), and 76.8% of White patients versus 74.4% of AI/AN patients had chemotherapy for Stage IV disease (*p* = 0.71). Lastly, regarding radiation therapy, 5.5% of White patients versus 5.0% of AI/AN patients had radiation therapy for Stage I disease (*p* = 0.06), 19.6% of White patients versus 22.1% of AI/AN patients had radiation therapy for Stage II or III disease (*p* = 0.31), and 9.4% of White patients versus 9.4% of AI/AN patients had radiation therapy for Stage IV disease (*p* = 0.38). Note that for the population as a whole, 62.4% of patients had an unknown status of radiation therapy.

### 3.4 Survival Analysis

**FIGURE 3:**
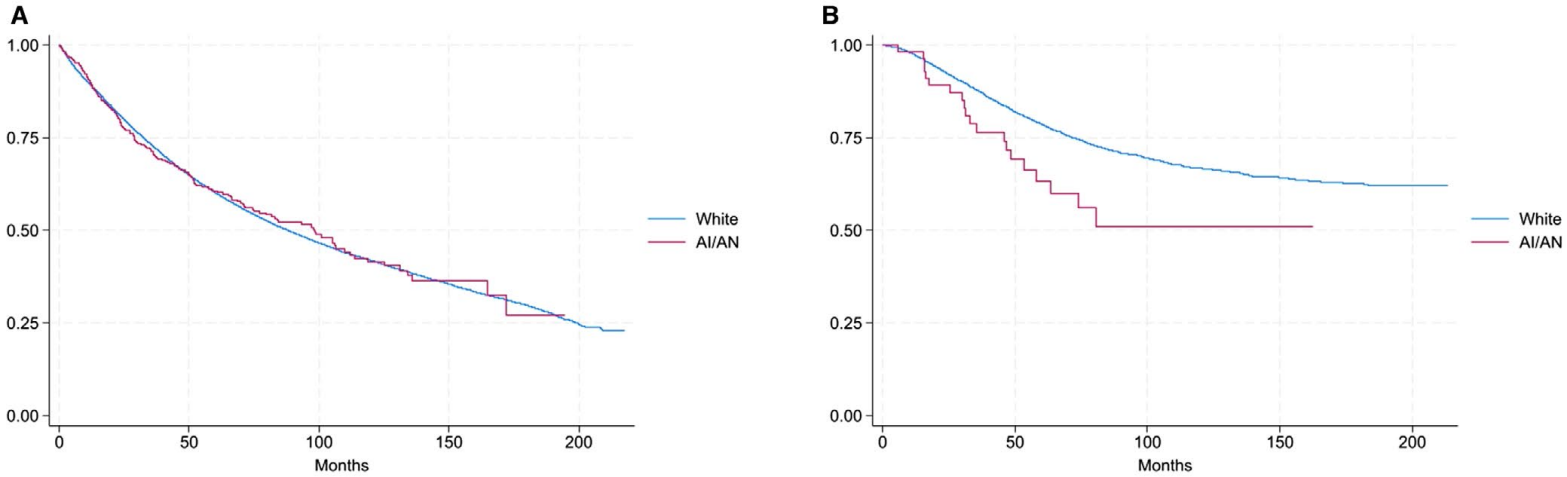
Overall survival (OS) by race categories stratified by age at diagnosis. (A) Age at diagnosis 45 years old or older (*p* = 0.98) (B) Age at diagnosis 44 years old or younger (*p* < 0.01). Kaplan–Meier curves displaying OS for patients with rectal adenocarcinoma. AI/AN, American Indian/Alaska Native.

As described above, the survival analysis was limited to Stages II and III. A total of 83,678 patients were included: 83,144 White patients and 534 AI/AN patients. Kaplan–Meier curves were used to assess OS stratified by age of diagnosis (Figure 3). In the population diagnosed before the age of 45, the AI/AN population had a steeper slope leading to the separation of the curves, consistent with a lower probability of OS for this population (Figure 3B; log‐rank *p* < 0.01). However, no difference was noted in the curves for patients diagnosed after age 45 (Figure 3A; log‐rank *p* = 0.98). Results of the multivariate Cox‐proportional hazards model with associated covariates are included in Table 2. Adjusted analyses suggested an increased hazard of death for AI/AN patients in comparison to White patients (HR, 1.14; 95% CI, 1.05–1.25; *p* = 0.003).

**TABLE 2:** Results of multivariate Cox‐proportional hazard model among 83,678 patients with rectal cancer stages II and III at diagnosis.

	Hazard ratio	95% Confidence interval	*p*
Race			
White	Reference		
AI/AN	1.14	1.05–1.25	0.003
Age	1.04	1.04–1.04	< 0.001
Sex			
Female	Reference		
Male	1.17	1.16–1.19	< 0.001
Stage			
Stage II	Reference		
Stage III	1.28	1.27–1.30	< 0.001
Charlson‐Deyo score			
Score 0	Reference		
Score 1	1.26	1.24–1.28	< 0.001
Score 2	1.45	1.41–1.49	< 0.001
Score 3+	1.97	1.89–2.05	< 0.001
Facility type			
Community cancer program	Reference		
Comprehensive community cancer program	0.93	0.90–0.95	< 0.001
Academic/research program	0.79	0.77–0.82	< 0.001
Integrated network cancer program	0.90	0.87–0.93	< 0.001
Unknown	1.40	1.32–1.48	< 0.001
Urban/rural			
Metro	Reference		
Urban	1.07	1.05–1.09	< 0.001
Rural	1.07	1.03–1.12	0.002
Unknown	0.79	0.76–0.83	< 0.001
Insurance			
Not insured	Reference		
Private insurance	0.62	0.60–0.64	< 0.001
Medicaid	1.00	0.95–1.04	0.87
Medicare	0.77	0.74–0.80	< 0.001
Other government (IHS)	0.72	0.67–0.77	< 0.001
Unknown	0.64	0.60–0.68	< 0.001
Surgical treatment			
Surgery	Reference		
No surgery	2.38	2.24–2.42	< 0.001
Unknown	1.47	1.23–1.45	< 0.001
Chemotherapy			
Chemotherapy	Reference		
No chemotherapy	1.66	1.63–1.69	< 0.001
Unknown	1.63	1.55–1.71	< 0.001
Radiation therapy			
Radiation	Reference		
No radiation	1.01	0.98–1.04	0.49
Unknown	1.43	1.39–1.46	< 0.001

*Note:* Statistical significance set at *p* < 0.05. Multivariable analysis with Cox‐proportional hazard models with Breslow method for ties.

Abbreviations: HSD, High School Diploma; IHS, Indian Health Services.

## 4 Discussion

The heterogeneous AI/AN community presents unique health challenges, largely influenced by historical and systemic factors leading to disparities in healthcare access and socioeconomic status [2, 3, 4, 26]; such factors affect healthcare utilization [2, 3, 4]. In our study, one of the most notable findings is the younger age at rectal cancer diagnosis in the AI/AN population compared to the reference White population; AI/AN individuals were diagnosed almost 5 years earlier—on average, with AI/AN patients being diagnosed at age 59.9 years, compared to 64.5 years for White patients. The younger age at diagnosis of colorectal cancer among AI/AN individuals compared to White counterparts has been reported in previous research [5] and highlights a critical need for understanding unique risk factors faced by the AI/AN community and implementing culturally tailored early intervention strategies. It is further concerning to see a trend toward worse survival in the under 45 age cohort, albeit in an unadjusted assessment. The mean age of the AI/AN population, being younger than the age of onset for rectal cancer in the general US population (age 63.5 [13]), implies that early‐onset cancer might be related to the particular demographic and environmental characteristics and exposures of these populations, highlighting the importance of ensuring adequate access to screening and patient education [27, 28, 29, 30, 31, 32], and targeting community outreach to higher risk populations [27]. Similarly, we found a higher hazard of death among AI/AN patients with rectal cancer despite adjusting for demographic, socioeconomic, clinical, and treatment factors, warranting further research and intervention.

Studies have shown that decreased utilization of cancer screening within minority populations can be attributed to barriers such as inadequate health insurance [28], language differences, and lower health literacy [27, 28, 29, 30]. Over one‐third of the AI/AN population in our study lived in the lowest income quartile catchment areas, and nearly one‐third resided in areas of lower high school completion rates, which are factors known to correlate with worse outcomes due to reduced access to consistent care and preventative cancer screening utilization [1, 33]. In addition, the Indian Health Service (IHS) plays a crucial role in providing CRC screening for the AI/AN population. However, tribal health centers may face barriers such as providers' knowledge gaps on appropriate CRC screening intervals [34], limited use of reminder alerts on electronic health records [35], shortage of trained providers to conduct follow‐up colonoscopies [34], and limited funding to contract services not available in the IHS healthcare facilities [2, 4, 34]. Thus, exposure to lifestyle risk factors commonly associated with lower socioeconomic status and inadequate access to CRC screening may also contribute to earlier onset of rectal cancer and more advanced stages at diagnosis in these populations [6].

All patients included in this study were treated in CoC‐accredited facilities, which each maintain a high level of quality. When looking at differences in receipt of treatment between the AI/AN and White populations, we see fairly similar rates of treatment, though more AI/AN patients did receive chemotherapy in early stages compared with their White counterparts. We note that when keeping covariates and treatment factors steady (i.e., same level of treatment), we see a higher hazard of death for AI/AN patients. These findings suggest that, beyond treatment and the included covariates, other factors not evaluated in this study are contributing to survival differences. Perhaps structural socioeconomic factors such as systematic racism, challenges with transportation, food security, distance to healthcare facilities, and literacy affect this outcome via both the utilization of preventive health services and cancer treatment compliance adherence [4, 27, 36].

The insights from our study call for comprehensive interventions tailored to AI/AN populations. Strategies must include not only clinical efforts but also address broader socioeconomic and cultural contexts to improve healthcare access, participation in screening programs, and, ultimately, health outcomes. There is a crucial need for policy measures and health system reforms that ensure culturally competent care and equitable resource allocation to these underserved communities [4, 27, 33, 34].

Our study is not without limitations, like challenges faced by other NCDB‐based studies, such as its retrospective design, potential for missing data, and possible unmeasured confounding bias. Efforts were made to mitigate these through statistical methods, yet the possibility of undercounting AI/AN individuals remains, as not all facilities may identify AI/AN as a distinct population. Prior research reported on the low coverage of the AI/AN population in the NCDB [15], which affects the ability to generalize our results to AI/AN communities not represented in this database. Further, there are statistical limitations of having a small exposed (AI/AN) population compared to a much larger reference (White) population. This highlights the importance of accurate data capture and the need for community‐engaged research approaches to understand and address the disparities fully. The survival differences among stages might be because of factors not evaluated in this study, such as more intensive treatment or better response among different racial groups. As the study includes only individuals treated at CoC‐accredited facilities, it is not possible to generalize the findings to the broad population of AI/AN patients with rectal cancer who may have received treatment in non‐CoC‐accredited facilities.

## 5 Conclusions

AI/AN patients presenting to CoC‐accredited facilities are diagnosed with rectal cancer at a younger age, more advanced stage, and have worse OS when compared with White counterparts. This disparity is indicative of a deeper, systemic issue that may include lifestyle factors, environmental exposures, and socioeconomic barriers affecting healthcare access and utilization [2, 3, 4].

Our findings underscore the urgency for targeted interventions to mitigate these disparities. Future efforts must be directed toward fostering community awareness, enhancing cancer screening programs, improving health‐seeking behaviors within AI/AN communities [37, 38], and maintaining standards of practice for this at‐risk group [3, 39]. Such efforts should be guided by the values and cultural contexts of the AI/AN communities to ensure that they are culturally sensitive and effective [9, 27, 37, 38].

Partnerships between community groups and academic institutions will be crucial in devising strategies to overcome structural barriers and increase the utilization of cancer screening [4, 37]. Tailored outreach programs that respect and incorporate traditional Native North American and Alaskan Native beliefs and practices are essential for encouraging early diagnosis and intervention, which could significantly improve prognosis and survival rates.

The complexities of healthcare provision to North American Indigenous communities, including factors such as rurality, cultural diversity, and socioeconomic challenges, call for a multifaceted approach. Policies must be formulated to not only improve healthcare delivery but also to enhance living conditions and educational and employment opportunities for all populations, thus addressing the root causes of health inequities.

## Author Contributions

Study concept and design: Broc S. Kelley, John M. Hampton, Margaret R. Walker, Andrea Schiefelbein, Roberto J. Vidri, Noelle K. LoConte. Data acquisition: Broc S. Kelley, Margaret R. Walker, Noelle K. LoConte. Data analysis: Broc S. Kelley, John M. Hampton, Noelle K. LoConte. Data interpretation: All authors. Writing – original draft: Broc S. Kelley. Writing – review and editing: All authors. Final approval of manuscript: All authors.

## Ethics Statement

This study was conducted with the analysis of secondary de‐identified data and did not require Institutional Review Board approval. Researchers did not have access to any links that could connect the study data to the patients from whom the data descended.

## Conflicts of Interest

Noelle LoConte receives unrestricted research funding from Exact Sciences.

## Supporting information


Figure S1.

## Data Availability

The NCDB is an oncology outcomes database for more than 1500 CoC‐accredited cancer programs in the United States and Puerto Rico. The Health Insurance Portability and Accountability Act (HIPAA)‐compliant Participant User Data Files (PUFs) with de‐identified data utilized in this study are available through an application process to investigators associated with CoC‐accredited cancer programs. NCDB, CoC, and the American College of Surgeons are not responsible for the statistical validity of the data analysis or the conclusions drawn by the authors. Data are available at https://www.facs.org/quality‐programs/cancer‐programs/national‐cancer‐database/.
