# Supplementary material for: Rectal Cancer Disparities Among the American Indian/Alaskan Native Populations
Source: Cancer Med. 2025 Apr 19;14(8):e70892. doi: 10.1002/cam4.70892 (PMC12008663; doi:10.1002/cam4.70892)
Supplement: Supplementary file 1 — Figure S1. [file CAM4-14-e70892-s001.docx]

**Supplemental Figures**

**Figure 1.** Directed Acyclic Graph detailing covariate relationship assumptions for survival analysis and choice of variables for multivariate survival modeling.


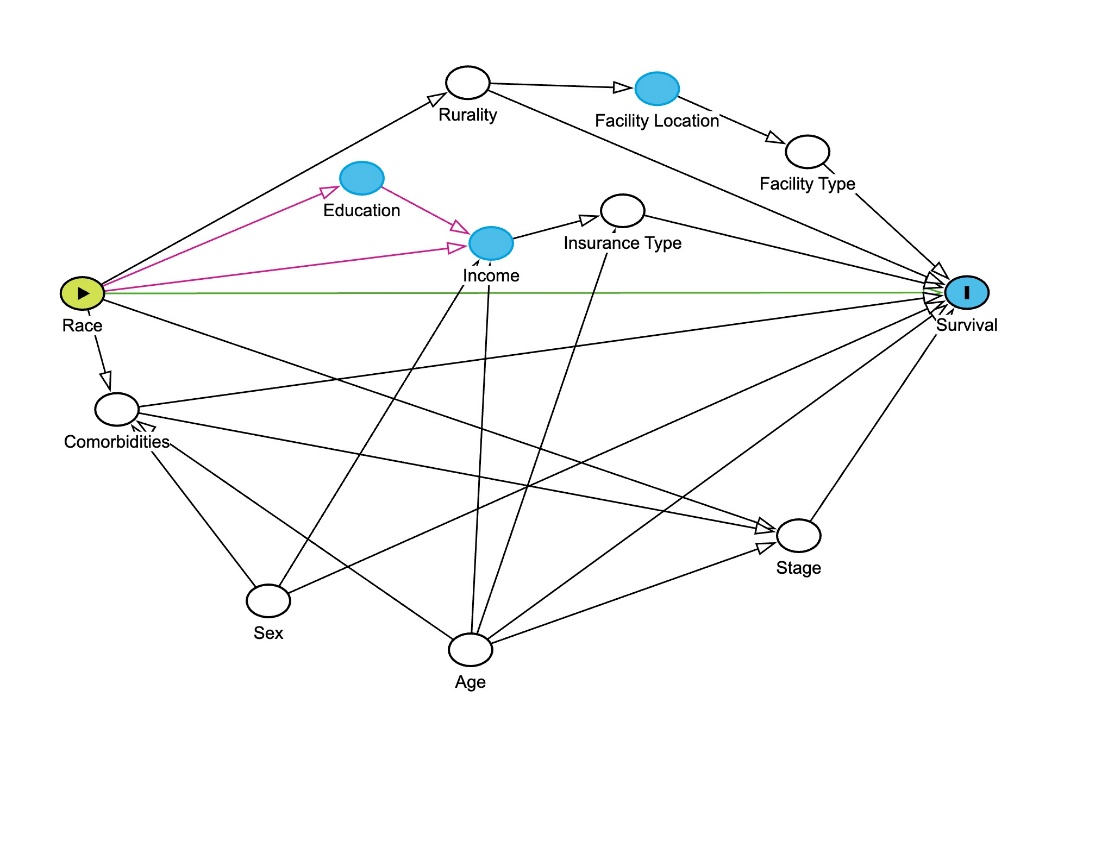

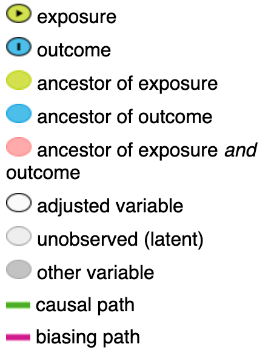


**Figure 2**. Boxplot displaying age at diagnosis by race


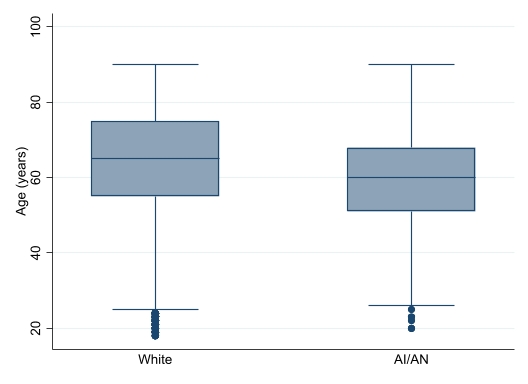


Legend: Gray central lines represent the median values (50th percentile), x represent mean value, while the boxes contains the 25th to 75th percentiles of each group. AI/AN= American Indian /Alaska Native; NHW= Non-Hispanic White.
